# Prevalence of Dietary Behavior and Determinants of Quality of Diet among Beneficiaries of Government Welfare Assistance in Poland

**DOI:** 10.3390/ijerph16030501

**Published:** 2019-02-11

**Authors:** Sylwia Kałucka, Dorota Kaleta, Teresa Makowiec-Dabrowska

**Affiliations:** 1 Department of Hygiene and Epidemiology, Medical University of Lodz, 90-647 Lodz, Poland; dkaleta@op.pl; 2 Department of Work Physiology and Ergonomics, Nofer Institute of Occupational Medicine, 91-348 Lodz, Poland; tmd@imp.lodz.pl

**Keywords:** dietary behavior, Dietary Quality Score, disadvantaged groups, low socio-economic status population

## Abstract

Diet, as a modifiable factor for good health maintenance, reduces the risk of numerous non-communicable chronic diseases and prevents premature death. The aim of the study was to examine the prevalence of a dietary behavior and to find out what the determinants of diet quality among the low socio-economic status population are. The studied sample consisted of 1710 respondents. Only 3% of the beneficiaries had healthy dietary habits. Unhealthy dietary habits dominated in all the study group regardless of the subjects’ level of education (*p* < 0.001). Higher odds of unhealthy dietary habits were reported among the respondents with primary, vocational, and secondary education than among the respondents with high education (for the primary education OR = 11.10; 95% CI: 5.86–21.01; *p* ≤ 0.001; for vocational education OR = 10.54; 95% CI: 5.79–19.18; *p* ≤ 0.001 and for secondary education OR = 5.83; 95% CI: 3.48–9.79; *p* ≤ 0.001). The unhealthy dietary behavior prevalence among beneficiaries of government welfare assistance in Poland is much higher than in the general population. Since only educational level is a determinant which is significantly correlated with the unhealthy dietary behavior, promotion of a healthy diet among disadvantaged individuals should be focused on this factor.

## 1. Introduction

A healthy diet has considerable impact on our well-being and on our health.

Currently, a lot of people have an ‘unhealthy’ diet and a low physical activity, which are the leading causes of major non-communicable chronic diseases in developed and developing countries across the world [1]. There is no doubt, as indicated by numerous studies, that a healthy diet based on fruit and vegetables, fish, and not many meat products, and its high quality are associated with the lower incidence of hypertension, cholesterol problems, coronary artery disease, type 2 diabetes, stroke, and certain cancers as well as other health benefits [2,3,4]. According to the World Health Organization (WHO), the growing burden of non-communicable diseases substantially contributes to the global burden of the disease, premature death, and disability [5]. 

Now it is important what kind of a diet is used among a population, including the ratio of individual components of the diet and frequency of their consumption daily or weekly (consumption of saturated and unsaturated fats and cholesterol, a rich or poor in fruit and vegetables diet, refined carbohydrates). If components of an ‘unhealthy’ diet dominate, it directly affects higher mortality rates and lower life expectancy in such a population. Diets, such as Mediterranean or vegetarian ones are related to beneficial health effects. These diets are characterized by low meat intake (low saturated fat and cholesterol), higher intake of fruit, vegetables, and whole-grain products. They clearly reduce the risk of coronary heart disease (CHD), obesity and type 2 diabetes [6]. Many epidemiologic and clinical trial data strongly support the fact that dietary patterns rich in fruit, vegetables [7], whole grains, and nuts can reduce the risk of CHD—one of the most common non-communicable chronic diseases [8,9]. World Health Organization and Food and Agriculture Organization of the United Nations (FAO) recommend consumption of at least two servings of fruit and three servings of vegetables per day [7,10]. Despite the fact that these recommendations are repeated in majority of nutritional guidelines of scientific societies concerning prevention of non-communicable chronic diseases, a large part of the global population does not achieve these targets. Research among a large population of Austrian adults (*N* = 15,474) has indicated that subjects on a healthy diet (a diet rich in fruit and vegetables) more often belonged to a higher socio-economic status (SES) group than to a low SES one [9]. In the low SES subjects with a diet rich in meat products, a higher number of chronic conditions, higher body mass index (BMI), an enhanced vascular risk as well as lower quality of life have been observed [9]. Barriers to following those nutrition recommendations can be related to high prices and unavailability of healthy food especially for the low socio-economic status population. In the developed nation of Canada, food price is the strongest determinant for choosing a particular type of food for the low SES group. Higher income determines a healthier diet among Canadians. Improved nutrition may be difficult to achieve for low-income SES Canadians because there are economic and cultural thresholds [11]. Other barriers for them are: family members preferences, convenience of unhealthy foods, and inability to break lifelong habits [12].

It is extremely important to get to know a dietary behavior and determinants of quality of the diet among disadvantaged groups well, because in almost all the countries, the rates of death and poorer self-assessments of health have been substantially higher in groups with a low SES than in those with a higher SES [13,14].

The cross-sectional cohort studies in Eastern, Central, and Western European populations (*N* = 21,326) have shown that consumption of seven predefined healthy food products (i.e., fruit, vegetables, wholegrain bread, vegetable-fat spread, vegetable cooking fat, low-fat milk, and low-fat cheese) was positively associated with higher education, occupational position and fewer economic difficulties [15]. There are many differences in the associations between the socio-economic indicators and healthy food habits in the countries presented in this study. That is why factors influencing the prevalence of dietary behavior and its determinants should be recognized separately in each country and socio-economically diverse subpopulations (with special attention paid to low SES). There is a lack of studies that would compare the diet quality among the low socio-economic status population in Poland. 

Another factor that influences the diversity and quality of a diet is related to its seasonal or all-year availability. Observation of women living in the rural South Asia (the Terai of Nepal, *N* = 15,899) has proven that the women belonging to a low SES group consumed rice, potatoes, legumes and vegetable oil regularly but they consumed animal products and fruit and vegetables less frequently than weekly. Most foods (whether available all year-round or seasonally), were eaten more often by women with higher socio-economic standing and higher education (literacy). A diet in higher SES groups was more diverse and included majority of the food-groups (including fruit and vegetables) in comparison with a diet in the low SES groups [16]. Food insecurity is associated with poor health consequences in the low SES population (especially among women of reproductive age) and it may be associated with an increased risk of chronic non-communicable diseases [16,17]. Studies published in the last decade of the 21st century support significance of the level of education as the strongest factor influence influencing on perception of a healthy diet, which the study carried out in 15 countries of the European Union demonstrated at the end of the 20th century [18]. A multicenter prospective cohort study performed in Italy among over 45,000 subjects has shown a positive relationship between higher education and healthy dietary habits (reduced consumption of processed meats, sweet beverages, and increased consumption of fruit, vegetables, fish, and olive oil) [19]. Similarly, higher scores for Heathy Eating Index, Mediterranean Diet Score, and Healthy Dietary Pattern among subjects (1852 military men) were associated with higher education and income [20].

Socio-economic inequalities in dietary healthy behavior tend to appear in the early stages of life before entering adulthood. The influence of education in the low socio-economic status population is present from the youngest age—higher prevalence of unhealthy eating habits among children and adults with a lower level of education is observed more frequently [21,22]. Then those unhealthy dietary patterns are continued into primary school [23], in 10-year-old children more frequent unhealthy eating behavior is associated with a decreased BMI in low SES population [24]. Negative dietary (especially skipped skipping meals, lower consumption of fruit and vegetables) observed among teenagers with a low SES, results in the higher prevalence of obesity among them [25]. Children of the parents with a low SES most often finish their education at the level of secondary school. Therefore, the knowledge they gain during their education serves them also in their adulthood. Thus, it is worth investing in school education and continuing raising the state of awareness on healthy eating habits also in adults because then knowledge on healthy eating increases along with age [26]. In the low SES middle-aged and elderly-aged people who start developing non-communicable chronic diseases [26,27], their low level of knowledge on a healthy diet is connected with poorer diet quality [28] and poorer adherence to dietary guidelines [29].

To assess if a diet of a given in population is healthy, the number of products consumed daily, weekly, the type of products and the quality (variety) of such a diet should be taken into account. The most important food components that are part of a healthy diet are: fats, vegetables, fruit, and fish. There are some diet-quality scores, that are used across the world in many studies in order to show dietary behavior, specific eating patterns and adherence to a healthy diet [30]. Higher scores of all Dietary Quality Score (DQS) are associated with a lower body mass index, lower waist-to-height ratio and waist circumference, which is also useful in assessment of the risk of non-communicable chronic diseases (NCDs). The diet quality scores have been used to indicate a factor influencing childhood overweight and obesity [31], or to assess the dietary quality in adults [32]. The tool that is most suitable for our study, is the dependent variable DQS. Dietary Quality Score is a simple tool and questions included in it are easy to answer. It was changed by the authors in the past [33]. It is the most compatible with our study and has been also modified for Polish study population. Dietary Quality Score concerns four food components that define a healthy diet: vegetables, fruit, fish, and fat (cooking and spread). It allows, after summing up the points, determination of what kind of a diet the subjects have: healthy, average, or unhealthy. 

Once reduction of cigarette smoking in the contemporary world takes place it is the unhealthy dietary behavior, next to the lack of physical activity, that will become one of the most important risk factors of many NCDs. What is more, a diet is a modifiable risk factor for these diseases and an improper dietary behavior can be changed into a healthy one at any age.

### Study Aim

Factors influencing prevalence of a dietary behavior and determinants of quality of the diet among beneficiaries of government welfare assistance have not been well-explored in Poland yet. Therefore, to help the beneficiaries of government welfare assistance in Poland, we have to get to know determinants of unhealthy diets and barriers to being on a healthy one.

## 2. Material and Methods

### 2.1. Characteristics of the Examined Region

The United Nations Development Program (UNDP) with its headquarters in New York operates in 166 countries, where it helps to solve national development problems. Based on the analysis in Poland, regions requiring support of the institutions of Social Assistance were selected. Poland, according to administrative division, has 16 voivodeships in which in total there are 314 counties. In Central Poland there is Piotrkowski district belonging to the Lodz voivodeship. This county was chosen by the UNDP after a performed analysis as it had a low Local Human Development Index. In the analysis of UNDP, the percentage of individuals using social assistance—an indicator of social development—and monthly income were taken into account. It turned out that, among 314 counties, it is in Piotrkowski district that has 9% of its residents requiring social assistance [34] and that this county has a low index of social development. This qualified it for the 11th place in Poland [35]. Based on qualifications concerning monthly income in the Social Assistance Act, it appeared that in this region monthly income per a person amounts to 634 PLN, and 514 PLN per member of a family, which allows inclusion of those individuals to a subpopulation with a low SES [34,35]. The UNDP analysis also included the state of health of a population. It appeared that the mortality index in Piotrkowski district was the highest in all 156 counties and that main causes of death in this region were chronic non-communicable diseases [14,36,37]. And so, the Health Index (HI) for the whole population of Lodz voivodeship amounted to 31.48 and was higher than that for the selected Piotrkowski county—HI—26.50 [36]. The data mentioned above resulted in the Polish government and the Norwegian Directorate of Health Affairs implementing a project entitled “Reducing Social Inequalities in Health”—Project PL-13 [38]. The aim of the project for the years 2009–2014 was to fight social inequalities among others through improvement of the state of health among low SES groups as part of a project entitled “Your Heart is Your Life”. 

### 2.2. Characteristics of the Study Sample

The study included 3636 individuals of those 11,867 who permanently used social assistance from Piotrkowski district [34]. The study was conducted in two stages. First, a list of people using the local government welfare assistance institutions was obtained. The individuals who agreed to take part in the study gave their informed written consent. The second stage consisted in presenting assumptions of the study and obtaining a permission of the Ethics Committee at the Medical University of Lodz, where the study was conducted. The study received a number RNN/243/15/KE. Having completed the above-mentioned requirements, the final study began. It lasted from October 2015 to February 2016. A face to face survey study, performed by trained pollsters, included 3636 adult people aged 18–59, who fulfilled the study inclusion criteria. The monthly income per person was the study inclusion criterion. In the case of beneficiaries of social assistance from Piotrkowski district, the income did not exceed 158 USD (634 Polish zloty) per month in the case of a person living alone and 128 USD (514 Polish zloty) in the case of a person living with a family. Refusal to agree in writing to take part in the study and not providing all answers to the questions included in the questionnaire prepared for the purpose of the study constituted exclusion criteria. Collected in such a way, research material was sent for the purpose of statistical analyses.

### 2.3. Characteristics of the Participants’ Survey

The study used a questionnaire containing socio-demographic data. Questions included in it concerned age of the respondents, their sex, education, and status of employment. Due to the fact that the study covered low SES groups the questionnaire contained questions on the subjective assessment of monthly income, subjective assessment of living conditions, cohabitation with a partner or living alone. The second part of the study concerned assessment of a dietary behavior and quality of a diet used by the study subjects. For this purpose, a verified DQS was applied, which was modified to the needs of the study. Dietary Quality Score characteristics based on Toft et al. and modified for Polish needs [33]. The questionnaire contains the most important four dietary components—i.e., vegetables, fruit, fish, and fat—in accordance with WHO recommendations, and responsible for the risk of chronic non-communicable diseases. It is estimated that frequency of eating and intake of nutrients in proper portions is associated with a healthy lifestyle [39]. Questions from the questionnaire concern the frequency of the consumed vegetables, fruit, fish, and fat (Table 1). A point is attributed to each dietary component. And so, consumption of >5 portions of vegetables a day receives 2 points, 2–5 receives1 point, and below 2 portions a week, 0 points. In the case of fruit, the frequency of consuming fruit a day is assessed. And so, those consuming more than three portions a day receive 2 points, three portions a week but no less than two a day receive 1 point, below three portions a week get 0 points. A portion of fish above 200 g a week gets 2 points, less than 200 g a week 1 point, and no fish at all gets 0 points. Assessment of fat is divided into fat spreads and cooking fats. No fat gets 0 points; vegetable margarine, 1 point; and butter, blended spread, lard get 0 points. Then the obtained points for fat are summed up. The more points a study subject gets the more often he/she consumes fruit, vegetables, fish and the more seldom he/she uses fat such as butter for cooking or frying. Then, 7–8 points mean that a subject can be qualified as the one with healthy dietary habits. Less often consumed vegetables, fruit and fish are characteristic of an average diet, while a low consumption of vegetables, fruit, and fish results in receiving 0–3 points which qualifies a subject as the one with an unhealthy diet (Table 2). 

### 2.4. Characteristics of the Statistical Analyses

To statistical calculations, chi-square test and the univariable and multivariable logistic regression was used. The study population was divided into four subgroups and an extended Mantel–Haenszel χ^2^ test was used to statistically analyze these groups. The odds ratios (ORs) and the 95% confidence interval (CI) of each indicator in relation to an unhealthy dietary behavior showed by using the univariable and multivariable logistic regression analyses. The variables used to adjust the models were: gender, age, the level of education, employment status, subjective assessment of monthly income, subjective assessment of living conditions, cohabitation with a partner and/or a family. 

All *p*-values were two-sided and *p* < 0.05 was used to indicate the statistically significant. The whole statistical analysis was performed by the use of the STATISTICA Windows XP version 10.0 program (StatSoft Poland Inc., Tulusa, OK, USA). 

## 3. Results

The study involved 3,636 beneficiaries of social assistance. Those whose surveys were missing some information were eliminated from the study. In the end, 1,710 subjects were included in the final statistical analysis (response rate = 47%). Socio-demographic characteristics of the study population are presented in Table 3 and Dietary Quality Score of the study participants in Table 4. 

### 3.1. Socio-Demographic Characteristics of the Study Population

Of the total 1,710 respondents, 66.8% were females (*n* = 1142) and 33.2% were males (*n* = 568) with the mean age of 39.2 ± 7.7 years (38.2 ± 7.2 for the females and 41.1 ± 8.1 years for the males; *p* < 0.001) (Table 3). Socio-demographic characteristics of the study population also contain data on the education, employment status, subjective assessment of monthly income, subjective assessment of living conditions, cohabitation with a partner/family or living alone (Table 3). 

### 3.2. Dietary Quality Score Characteristics among the Study Population

The smallest group (only 3.0%) of the study population were beneficiaries who had healthy dietary habits (Table 4). There was also no statistically significant difference between the women (3.5%) who had healthy dietary habits and the men (2.1%) (*p* > 0.05). The majority of the respondents (90.7%) had unhealthy dietary habits. There was no statistically significant difference between the women (89.7%) and the men (92.4%) who had unhealthy dietary habits (*p* > 0.05) too.

The respondents were divided into four age groups: under 30, between 30–39, 40–49, and 50–59. There were no statistically significant differences regarding healthy dietary, average dietary, and unhealthy dietary habits and among these four categories of age groups (*p* > 0.05).

Further data show the level of education and frequency of the occurrence of healthy, average and unhealthy dietary habits. Most frequently healthy dietary habits were presented by the subjects with a high level of education—one-fourth of them (26.9%). Average dietary habits were represented by 11.8% of the respondents. The subjects with a primary level of education had unhealthy dietary habits most often (94.2%), those with vocational education (93.6%), secondary level of education (89.5%), and the least often unhealthy dietary habits were reported among a high level of education group (61.3%) (*p* < 0.001). Unhealthy dietary habits dominated in all the study groups regardless of the study subjects’ level of education (*p* < 0.001). Only single subjects with a primary level of education—3 (0.6%), vocational—9 (1.6%), secondary level of education—15 (2.6%) had healthy dietary habits (*p* < 0.001).

There was no statistically significant difference between the employment status and three types of healthy dietary habits (*p* > 0.05). There was no statistically significant difference between the subjective assessment of monthly income, subjective assessment of living conditions, cohabitation with a partner and/or a family and healthy/average/unhealthy dietary habits among the subjects (Table 4). 

Table 5 presents frequency of consumption of the most important food ingredients (vegetables, fruit, fish, fat). Three-fourths of the presented population consumed less vegetables than two servings per week (76.7%), one-third of them (33.1%) consumed fruit in an amount less than free pieces per week, almost all the subjects (90%) ate no fish, but often used fat spread (butter, blended spread, lard—61.5%) and cooking fat (margarine, butter—40.2%). 

Three-fourths of the study population (the females—75.8% and the males—78.5%), admitted to consuming less than 2 servings of vegetables weekly, only 12.1% of the females and 10.9% of the males 2–5 servings of vegetables weekly and 10.6% of the males and 12.1% of the females more than 5 servings. There was a statistically significant difference between the women and the men with regard to the frequency of consuming vegetables (Table 5). The women consumed three pieces of fruit daily more often when compared to the males—9.3% (*p* = 0.0089). 

Only 4.4% of the study population consumed more than 200g of fish per week, and the women ate it more often (5.3%) than the men (2.6%) (*p* = 0.0089). There was no statistically significant difference between the women and the men who did not eat fish (the female—89.2%, the males—91.6%) (*p* = 0.11). 

Fewer participants (5.6%) did not use any fat spread and cooking fat (2.1%). More often, the men gave up using fat spread (7.9%) and cooking fat (2.5%) compared to the women (4.5%) (*p* = 0.004) and (1.9%), respectively. More often the women used fat for bread (62.7%) than the men (59.0%) but cooking fat like margarine/butter was used by the men more often (41.5%) than the women (39.6%). There was no statistically significant difference between the women and the men in both cases (used fat spread and cooking fat) (*p* > 0.05). The participants of the study of both sexes used vegetable margarine such as fat spread (the women—32.8% and the men—33.1%) and such as cooking fat (the women—58.5% and the men—56.0%) with the same frequency and there were no statistically significant differences (*p* > 0.05) (Table 5).

The results of the logistic regression analyses for unhealthy dietary habits with socio-demographic characteristics was included in Table 5. The ORs and the 95% CIs for unhealthy dietary habits indicated that the men had much higher odds of unhealthy dietary habits compared to the women (OR = 1.35; 95% CI: 0.97–2.01; *p* ≤ 0.1). 

The level of education was an important factor when considering dietary habits. The ORs and the 95% CIs for unhealthy diet habits showed that the level of education played a significant role. The lower the education of the beneficiaries, the higher the odds of unhealthy dietary habits.

In the study, the respondents with primary education had 11-times higher odds of unhealthy dietary habits, those with vocational education had 10-times higher odds of unhealthy dietary habits, and those with secondary education had 5-times higher odds of unhealthy dietary habits than the respondents with a high level education (for the primary education OR = 11.10; 95% CI: 5.86–21.01; *p* ≤ 0.001; for vocational education OR = 10.54; 95% CI: 5.79–19.18; *p* ≤ 0.001and for secondary education OR = 5.83; 95% CI: 3.48–9.79; *p* ≤ 0.001).

The second significant outcome related to the association between the employment status and dietary habits. The subjects with a temporary job had more than two times higher odds of unhealthy dietary habits. The unemployed also had much higher odds of unhealthy dietary habits than the subjects with a permanent job (for the temporary job OR = 2.37; 95% CI: 1.10–5.07; *p* ≤ 0.05) (Table 6).

In the multivariable logistic regression, the analysis showed that only level of education is a significant impact on dietary habits.

## 4. Discussion

In the study, the authors estimated the prevalence of dietary behavior and found out about the factors which determined an unhealthy diet among adult beneficiaries of government welfare assistance in Poland. The results revealed that a small percentage of the beneficiaries had healthy dietary habits (while majority of them (both women and men) had unhealthy dietary habits. In the general population of Poland, the prevalence of a healthy diet is five times higher (15%) than among the present beneficiaries of the welfare group [40]. Sixty percent of adults Poles declared a low-quality diet, which is not a satisfactory result. Such a frequency of a low-quality diet is still higher than among our study disadvantaged group where a poor diet was present in the majority of the study subjects (90%) [40].

Additionally, the majority of the study subjects did not reach the healthy dietary recommendation for selected food ingredients. This concerns all components of a diet, including frequency of not eating vegetables which could be estimated thanks to the use of the DQS. Thanks to DQS we can clearly demonstrate if a given population observes healthy diet recommendations or has an average or unhealthy diet (diet that contributes to the development of non-communicable chronic diseases, neoplasms). Dietary Quality Score also allows showing which element of a diet is at a proper, insufficient, or low level of consumption. The study showed how important it is to use a verified and simultaneously simple in use tool assessing the diet quality. Not only does it specify a dietary behavior in a given population but also thanks to it we can elaborate targeted interventions that improve behaviors concerning those food components the consumption frequency of which does not fulfill recommendations. It is especially important for poor people who have tendency to associate healthier food products with higher prices [41]. In the study, among low SES population, men ate fruit less frequently during a day or a week than the women. There is evidence on social patterning in food motivation. Men with low income and a low level of education more rarely eat fruit [42] and vegetables [43]. The study among Dutch adults has also shown, that subjects with the lowest education chose fruit less often and adhered to the fish guideline less frequently [29]. In our low SES population, an inappropriate balance and frequency of consumption also concerned the fat. Both the women and men did not consume the recommended amount of fish (i.e., more than 200 g a week). The men ate fish considerably less frequently. Such a portion of fish weekly is sufficient for primary prevention of cardiovascular risk, reduces the risk of coronary death by 36% and total mortality by 17% and should be used by majority of the subjects [44]. Individuals with a low SES may not be aware of what type of a diet they should use in order to stay healthy. Our study subjects also chose an improper type of cooking fat too frequently, which is a significant component of an unhealthy diet. A study in Costa Rica has shown that health awareness and a high socio-economic status are strongly associated with the choice of unsaturated oil (proper cooking oil) in comparison with the palm oil, which is continuously used by people with the lowest SES [45]. Another systematic review and meta-analysis of randomized controlled trials emphasizes significant reduction of CHD events by the increased intake of polyunsaturated fat (PUFA) in place of saturated fat (SFA) [44]. In the study group, the intake of fat spread (butter, blended spread, lard) was almost twice higher than that of vegetable margarine. The men did not eat fat more often when compared to the women. An unhealthy dietary behavior, more frequent consumption of SFA fat, instead of PUFA, is associated with a high risk of development of neoplastic diseases of prostate in men [46,47] and breast cancer in women [48]. When using such a diet one can neither prevent development of non-communicable chronic diseases (NCDs) nor reduce the risk of premature death and disability. Among NCDs, coronary heart disease is the leading cause of mortality in the developed countries, but it is also a primary cause of death worldwide. Results of prospective cohort studies and clinical trials have indicated that there are three key dietary strategies which are effective in preventing coronary heart disease: increased consumption of omega-3 fatty acids from fish and plant sources; consumption of a diet rich in fruit, vegetables, nuts, and whole grains; and low consumption of refined grain products [49]. The diet of Polish adults in the general population is not properly balanced but it is still much better than among the adult beneficiaries of government welfare assistance. Poles’ dietary cholesterol and fruit/vegetables were consumed in recommended doses only by 44–80% of the respondents [40,50]. 

To modify unhealthy dietary habits into healthy ones, greater awareness of health benefits of a diet itself is needed as well as knowledge what a healthy diet is. In the presented study almost all the beneficiaries of welfare assistance presented an unhealthy dietary behavior. Statistical analysis of socio-demographic data and the diet used by the study subjects indicated, that it was only the education level that influenced a healthy dietary behavior. The respondents with primary, vocational, and secondary education, presented an unhealthy dietary behavior significantly more frequently than those with a high level of education. The subjects with a temporary job had a twice as high risk of unhealthy dietary habits as the subjects with a permanent job. However, that was not confirmed in the further multivariable logistic regression. Nutrition intervention programs should aim to learn not only about healthy foods that have health benefit, but also how to balance a healthy diet so that the cost is sustainable for people with low SES.

The remaining examined factors, such as age, gender, subjective assessment of monthly income, subjective assessment of living conditions, cohabitation with a partner or a family or living alone turned out to be insignificant determinants of making a choice concerning the quality of a diet by the studied beneficiaries of government welfare assistance in Poland. Similarly, in the described studies performed in European countries or among US-born Latinas and immigrants, it was the educational level—and not age or gender—that has had the strongest link to a healthy diet (fruit, vegetables) [15,18,26,29,51]. Kato et al. in the study among Japanese migrants to the United States, have presented that knowledge on an unhealthy diet, not genetic factors, will decide development of cardiovascular disease. Three Japanese populations living in Japan, Hawaii, and San Francisco after migration changed their diet and increased the intake of saturated fat as a percentage of total energy. After the diet change, the mean relative weight and serum cholesterol levels started to grow in parallel with the increased saturated fat intake [52]. Such unhealthy eating habits can be changed by raising the state of awareness of healthy eating habits, even in old age [26]. It is vital to take care of providing more sources of information on a healthy diet in order to maintain healthy eating habits. [18]. A study conducted by Fortmann S.P. et al. among Spanish-speaking and English-speaking participants has shown that even individuals with a low educational level who at the beginning of the study had higher dietary cholesterol and saturated fat could achieve better results thanks to education. After three years of the implemented educational program, in all studied groups the researchers received 20–40% decreases in dietary cholesterol and saturated fat. Positive changes referred to individuals both from the low SES and high-SES groups [53]. 

Identification of barriers of using a healthy diet will enable public health to construct efficient interventions referring to individual social subgroups (e.g., low SES) or the whole population. Interventions in the field of nutrition in the environment with a low socio-economic status should start from educating children from low SES families at school. Children of the parents with the lowest education have the worst results in terms of their knowledge on nutrition and healthy behaviors [54]. Introducing interventions at a younger age brings about effects in a form of dietary behaviors improvement (among others: increased consumption of fruit and vegetables, reduced amount of sweetened beverages) and thus, reduces the risk of obesity in the future [55]. During a three-year intervention in the field of nutrition of children from primary schools with a low socio-economic status (also including health promotion for the teachers and families), there was a two-fold increase in the improvement of following dietary recommendations (from 11% to 23% in the girls and from 12% to 23% for the boys) [56]. In that study, also an increase in physical activity of the pupils was reported (from 1% to 16% in the girls and from 3% to 7% in the boys). Education on a healthy lifestyle is significant, particularly for children from poor families as unhealthy dietary habits present in the childhood are continued in the adult life [57]. Those children do not have a chance to learn about proper dietary habits in their home environment because their parents do not have such knowledge [58]. Schools should educate not only in the field of science, but also in the field of a healthy lifestyle (including healthy dietary behaviors), which counteracts deepening social inequalities and gives opportunities to gain knowledge (often for life) to children from poor families [59]. Unhealthy dietary behaviors from childhood are continued into adult life [60,61], and even when people are very old [57,62]. This results in a worse health status in middle and elderly age [62], including development of non-communicable chronic diseases. This has been demonstrated by observations in the Republic of Serbia [63] as well as by 4- and 10-year studies in Italy and Greece, where high adherence to the Mediterranean diet has been reported among individuals with a higher socio-economic status, which directly contributed to a lower risk of development of cardiovascular disease (CVD), hypertension, and diabetes [60,61]. Socially disadvantaged groups are unaware of consequences of an unhealthy dietary behavior as a result of lack of knowledge and not understanding the risk of developing non-communicable chronic diseases. A study among African Americans with a low socio-economic status has indicated that among study subjects, one-quarter (24%) had diabetes, and a half of them (53%) suffered from hypertension and only as many as 12 % of them perceived their chronic kidneys disease (CKD) risk as high [12]. A cohort study in France (*N* = 11,931 men and *N* = 39,737 women) has revealed that better adherence to nutrition recommendations was inversely associated with a lower educational level [64]. We can change bad dietary patterns even in adults from poorer social groups, which has been proven by a 20-year educational program on healthy nutrition carried out among Australians. Thanks to it the largest positive changes have been reported among participants with a low socio-economic status. They gained the knowledge they needed on food categorization according to the frequency with which they should be consumed, planning the food budget, correct interpretation of nutritional values on food products. Thanks to education during this program, they were able to assess the relationship between a diet and obesity and a number of other diseases, so needed especially in the group with low socio-economic status [65].

Apart from education, as a factor that influences dietary behaviors among low SES groups to the largest extent, it is worth supporting those people using other interventions such as: vouchers for healthy food, family-based education, nutritional counseling, and group gatherings for individuals interested in making dietary changes [12].

Future efforts in public health should focus on better understanding of behaviors promoting healthy nutrition, education and raising awareness and effectiveness of healthy dietary habits in terms of a health status among beneficiaries of government welfare assistance.

Diet, as a modifiable factor for good health maintenance, reduction of the risk of numerous non-communicable chronic diseases, and prevention of premature death, will play a more and more significant role. It is even more significant, as thanks to promotion of education, we will be able to achieve a satisfactory effect even among individuals with a low SES. 

Adult beneficiaries of government welfare assistance who experience considerable decrease of the status of life, and their financial resources are sufficient only for basic needs in order not to choose the cheapest, often unhealthy products, should be not only supported financially but also educated in the field of healthy eating patterns and should be aware of their health benefits.

## 5. Strengths and Limitations of the Study

The present study has several strengths. Firstly, up until now, there have been no studies on dietary behavior among the socially disadvantaged population of social assistance beneficiaries from a rural district of Poland. 

Secondly, a large group of the beneficiaries aged between 18–59 (3636 persons) took part in the study and the final statistical analysis included 1710 subjects. The response rate was almost 50% of the average level compared to other questionnaire surveys conducted in Poland. 

Thirdly, a very important strength of the study is the fact that it examined the prevalence of a healthy dietary behavior but also looked for determinants of the quality of a diet.

Another strength of the study concerns the application of the useful Dietary Quality Score—used in other research—which enabled us to assess the quality of a diet of the study subjects. Finally, the results provide us with a basis to elaborate programs directly addressed to the examined group and not to the whole population, which will result in better outcomes of following the elaborated dietary recommendations. A limitation of the study is the fact that it focuses only on the current situation of the study subjects. We do not know what influenced having unhealthy dietary habits in the past—e.g., a household, family, school, neighborhood. Of course, one needs to have full awareness of the fact that in order to modify unhealthy dietary habits interventions in a form of education should be performed in the long run, repeated, verified, and the subjects should be motivated to maintain a good dietary habit and prevented from returning to bad habits.

Another limitation of the study refers to the questions used in the survey. The type of the questions used in the questionnaire has a nature of direct questions. There are no open questions, which would enable us to get to know the problem better and which could help in getting to know the reasons why the beneficiaries of government welfare assistance tend to have an unhealthy diet.

## 6. Conclusions

The prevalence of unhealthy dietary behavior among the beneficiaries of government welfare assistance in Poland is much higher than in the general population. What is interesting is that only one factor significantly correlates with the unhealthy dietary behavior in this socio-demographic group, and it is educational level. When starting to promote healthy dietary behavior and prevention of non-communicable diseases, we should focus on this factor and remember about substantial differences between the general population and the socially disadvantaged individuals.

## Figures and Tables

**Table 1:** Characteristic of Dietary Quality Score (DQS).

Food	Frequency	Score
Vegetables	>5 servings/week	2 points
	2–5 servings/week	1 point
	<2 servings/week	0 point
Fruit	>3 pieces/day	2 points
	>3 pieces/week and <2 pieces/day	1 point
	<3 pieces/week	0 point
Fish	>200 g/week	2 points
	<200 g/week	1 point
	No intake	0 point
Fat	None	2 points
Fat, spread	vegetable margarine	1 point
	butter, blended spread, lard	0 point
Fat, cooking	none/olive oil	2 points
	vegetable margarine, oil	1 point
	margarine/butter/blended spread/lard	0 point
Fat, summarized	6 points, summarized	2 points
	3–5 points, summarized	1 point
	2 points, summarized	0 point

**Table 2:** Dietary Quality Score categories.

Category	Score
Healthy dietary habits	7–8 points
Average dietary habits	4–6 points
Unhealthy dietary habits	0–3 points

**Table 3:** Characteristics of the study population *N* = 1710.

Variable	Total *N* = 1710	Men *n* = 568 (33.2%)	Women *n* = 1142 (66.8%)	*p*-Value
Age (years)				
<30	194 (11.3%)	47 (24.2%)	147 (75.8%)	*p* < 0.001
30–39	725 (42.4%)	201 (27.7%)	524 (72.3%)	
40–49	578 (33.8%)	211 (36.5%)	367 (63.5%)	
50–59	213 (12.5%)	109 (51.2%)	104 (48.2%)	
Education				
Primary	468 (27.4%)	204 (43.6%)	264 (56.4%)	*p* < 0.001
Vocational	566 (33.1%)	228 (40.3%)	338 (59.7%)	
Secondary	583 (34.1%)	128 (22.0%)	455 (78.0%)	
High	93 (5.4%)	8 (8.6%)	109 (91.4%)	
Employment status				
Permanent job	507 (29.6%)	215 (42.4%)	292 (57.6%)	*p* < 0.001
Temporary job	149 (8.7%)	70 (47.0%)	79 (53.0%)	
Disabled or retired	55 (3.2%)	28 (50.9%)	27 (49.1%)	
Unemployed	999 (58.4%)	255 (25.5%)	744 (74.5%)	
Subjective assessment of monthly income				
Sufficient to cover all living needs plus may save a certain amount	20 (1.2%)	4 (20.0%)	16 (80.0%)	*p* < 0.001
Sufficient to cover all living needs	188 (11.0%)	53 (28.2%)	135 (71.8%)	
Sufficient to cover basic needs only	894 (52.3%)	275 (28.7%)	619 (71.3%)	
Not sufficient to cover even the basic needs	433 (25.3%)	183 (42.3%)	250 (57.7%)	
Difficult to say	175 (10.2%)	53 (30.3%)	122 (69.7%)	
Subjective assessment of living conditions				
Fair	180 (10.5%)	58 (32.2%)	122 (67.8%)	*p* < 0.03
Rather fair	607 (35.5%)	173 (28.5%)	434 (71.5%)	
Neither fair nor poor	774 (45.3%)	284 (36.7%)	490 (63.3%)	
Rather poor	85 (5.0%)	28 (32.9%)	57 (67.1%)	
Poor	30 (1.7%)	14 (46.7%)	16 (53.3%)	
Difficult to say.	34 (2.0%)	11 (32.4%)	23 (67.6%)	
Cohabitation with partner and/or family				
No (living alone)	1444 (84.4%)	479 (33.2%)	965 (66.8%)	*p* > 0.05
Yes	266 (15.6%)	89 (33.5%)	177 (66.5%)	

**Table 4:** Categories of Dietary Quality Score and the study population.

Variable	Total	Healthy Dietary Habits		Average Dietary Habits		Unhealthy Dietary Habits	
	*N* = 1710	*n* = 52 (3.0%)	*p*-Value	*n* = 108 (6.3%)	*p*-Value	*n* = 1550 (90.7%)	*p*-Value
**Gender**							
Men	568 (33.2%)	12 (2.1%)	*p* > 0.05	31 (5.5%)	*p* > 0.05	525 (92.4%)	*p* > 0.05
Women	1142 (66.8%)	40 (3.5%)		77 (6.7%)		1025 (89.7%)	
**Age (years)**							
<30	194 (11.3%)	5 (2.6%)	*p* > 0.05	13 (6.7%)	*p* > 0.05	176 (90.7%)	*p* > 0.05
30–39	725 (42.4%)	27 (3.7%)		47 (6.5%)		651 (89.8%)	
40–49	578 (33.8%)	14 (2.4%)		38 (6.6%)		526 (91.0%)	
50–59	213 (12.5%)	6 (2.8%)		10 (4.7%)		197 (92.5%)	
**Education**							
Primary	468 (27.4%)	3 (0.6%)	*p* < 0.001	24 (5.1%)	*p* < 0.02	441 (94.2%)	*p* < 0.001
Vocational	566 (33.1%)	9 (1.6%)		27 (4.8%)		530 (93.6%)	
Secondary	583 (34.1%)	15 (2.6%)		46 (7.9%)		522 (89.5%)	
High	93 (5.4%)	25 (26.9%)		11 (11.8%)		57 (61.3%)	
**Employment status**							
Permanent job	507 (29.6%)	20 (3.9%)	*p* > 0.05	40 (7.9%)	*p* > 0.05	447 (88.2%)	*p* > 0.05
Temporary job	149 (8.7%)	3 (2.0%)		5 (3.4%)		141 (94.6%)	
Disabled or retired	55 (3.2%)	2 (3.6%)		2 (3.6%)		51 (92.8%)	
Unemployed	999 (58.4%)	27 (2.7%)		61 (6.1%)		911 (91.2%)	
**Subjective assessment of monthly income**							
Sufficient to cover all living needs plus may save a certain amount	20 (1.2%)	1 (5.0%)	*p* > 0.05	0 (0.0%)	*p* > 0.05	19 (95.0%)	*p* > 0.05
Sufficient to cover all living needs	188 (11.0%)	11 (5.8%)		17 (9.0%)		160 (85.1%)	
Sufficient to cover basic needs only	894 (52.3%)	18 (2.0%)		59 (6.6%)		817 (91.4%)	
Not sufficient to cover even the basic needs	433 (25.3%)	13 (3.0%)		21 (4.8%)		399 (92.2%)	
Difficult to say	175 (10.2%)	9 (5.1%)		11 (6.3%)		155 (88.6%)	
**Subjective assessment of living conditions**							
Fair	180 (10.5%)	4 (2.2%)	*p* > 0.05	14 (7.8%)	*p* > 0.05	162 (90.0%)	*p* > 0.05
Rather fair	607 (35.5%)	25 (4.1%)		39 (6.4%)		543(89.5%)	
Neither fair nor poor	774 (45.3%)	18 (2.3%)		46 (5.9%)		710 (91.7%)	
Rather poor	85 (5.0%)	3 (3.5%)		5 (5.9%)		77 (90.6%)	
Poor	30 (1.7%)	0 (0.0%)		1 (3.3%)		29 (96.7%)	
Difficult to say.	34 (2.0%)	2 (5.9%)		3 (8.8%)		29 (85.3%)	
**Cohabitation with partner and/or family**							
No (living alone)	1444 (84.4%)	45 (3.1%)	*p* > 0.05	96 (6.6%)	*p* > 0.05	1303 (90.2%)	*p* > 0.05
Yes	266 (15.6%)	7 (2.6%)		12 (4.5%)		247 (92.9%)	

**Table 5:** Frequency of consumption of the most important food ingredients in the study sample *N* = 1710.

Food	Frequency	Total (*N* = 1710)	Men (*n* = 568)	Women (*n* = 1142)	*p*-Value
Vegetables	>5 servings/week	198 (11.6%)	60 (10.6%)	138 (12.1%)	0.3616
	2–5 servings/week	200 (11.7%)	62 (10.9%)	138 (12.1%)	0.4672
	<2 servings/week	1312 (76.7%)	446 (78.5%)	866 (75.8%)	0.2135
Fruit	>3 pieces/day	210 (12.3%)	53 (9.3%)	157 (13.7%)	0.0089
	>3 pieces/week and <2 pieces/day	934 (54.6%)	290 (51.1%)	644 (59.4%)	0.0011
	<3 pieces/week	566 (33.1%)	225 (39.6%)	341 (29.9%)	0.0001
Fish	>200 g/week	75 (4.4%)	15 (2.6%)	60 (5.3%)	0.0104
	<200 g/week	96 (5.6%)	33 (5.8%)	63 (5.5%)	0.7994
	No intake	1539 (90%)	520 (91.6%)	1019 (89.2%)	0.1193
Fat Fat, spread Fat, Cooking	None minarine, vegetable margarine butter, blended spread, lard none/olive oil vegetable margarine, oil margarine/butter/blended spread/lard	96 (5.6%) 563 (32.9%) 1051 (61.5%) 36 (2.1%) 986 (57.7%) 688 (40.2%)	45 (7.9%) 188 (33.1%) 335 (59.0%) 14 (2.5%) 318 (56.0%) 236 (41.5%)	51 (4.5%) 375 (32.8%) 716 (62.7%) 22 (1.9%) 668 (58.5%) 452 (39.6%)	0.0041 0.9010 0.1387 0.4150 0.3244 0.0655

**Table 6:** Odds ratios (ORs) and 95% confidence intervals (CIs) for unhealthy dietary habits by sociodemographic characteristics.

Characteristics	Total *N* = 1710						
	Total	Unhealthy Dietary Habits		Univariable Logistic Regression		Multivariable Logistic Regression	
	*N*%	*N*%	*p*-Value	OR	95% CI	OR	95% CI
Gender							
Men	568 (33.2%)	525 (92.4%)	0.0737	1.39 ^#^	0.97–2.01	1.02	0.68–1.54
Women	1142 (66.8%)	1025 (89.7%)		Ref.		Ref.	
Age (years)							
<30	194 (11.3%)	176 (90.7%)	0.3652	Ref.		Ref.	
30–39	725 (42.4%)	651 (89.8%)		0.90	0.52–1.55	0.90	0.51–1.60
40–49	578 (33.8%)	526 (91.0%)		1.03	0.59–1.82	0.96	0.36–1.33
50–59	213 (12.5%)	197 (92.5%)		1.26	0.62–2.55	1.05	0.33–1.55
Education							
Primary	468 (27.4%)	441 (94.2%)	*p* < 0.001	10.32	5.82–18.27 ***	11.10 ***	5.86–21.01
Vocational	566 (33.1%)	530 (93.6%)		9.30	5.43–15.93 ***	10.54 ***	5.79–19.18
Secondary	583 (34.1%)	522 (89.5%)		5.40	3.29–8.87 ***	5.83 ***	3.48–9.79
High	93 (5.4%)	57 (61.3%)		Ref.		Ref.	
Employment status							
Permanent job	507 (29.6%)	447 (88.2%)	0.1403	Ref		Ref.	
Temporary job	149 (8.7%)	141 (94.6%)		2.37 *	1.10–5.07	1.99	0.90–4.39
Disabled or retired	55 (3.2%)	51 (92.8%)		1.71	0.60–4.91	1.58	0.53–4.76
Unemployed	999 (58.4%)	911 (91.2%)		1.39^#^	0.98–1.97	1.18	0.80–1.74
Subjective assessment of monthly income							
Sufficient to cover all living needs plus may save a certain amount	20 (1.2%)	19 (95.0%)	0.4076	2.45	0.31–19.38	3.50	0.40–30.42
Sufficient to cover all living needs	188 (11.0%)	160 (85.1%)		0.74	0.40–1.36	0.92	0.47–1.80
Sufficient to cover basic needs only	894 (52.3%)	817 (91.4%)		1.37	0.81–2.31	1.35	0.78–2.36
Not sufficient to cover even the basic needs	433 (25.3%)	399 (92.2%)		1.51	0.84–2.71	1.27	0.67–2.39
Difficult to say	175 (10.2%)	155 (88.6%)		Ref.		Ref.	
Subjective assessment of living conditions							
Fair	180 (10.5%)	162 (90.0%)	0.5320	Ref.		Ref.	
Rather fair	607 (35.5%)	543(89.5%)		0.94	0.54–1.64	0.88	0.49–1.58
Neither fair nor poor	774 (42.3%)	710 (91.7%)		1.23	0.71–2.14	1.03	0.51–1.70
Rather poor	85 (5.0%)	77 (90.6%)		1.07	0.45–2.57	0.97	0.25–1.70
Poor	30 (1.7%)	29 (96.7%)		3.22	0.41–25.18	2.29	0.28–18.92
Difficult to say	34 (2.0%)	29 (85.3%)		0.64	0.22–1.87	0.80	0.25–2.59
Cohabitation with partner and/or family							
No (living alone)	1444 (84.4%)	1303 (90.2%)	0.1773	Ref.		Ref.	
Yes	266 (15.6%)	247 (92.9%)		1.41	0.85–2.32	1.52	0.90–2.55

^a^ Fully-adjusted model including all statistically significant characteristics; ^#^ *p* ≤ 0.1; * *p* ≤ 0.05; ** *p* ≤ 0.01; *** *p* ≤ 0.001.
